# A case study of *Desmozoon lepeophtherii* infection in farmed Atlantic salmon associated with gill disease, peritonitis, intestinal infection, stunted growth, and increased mortality

**DOI:** 10.1186/s13071-017-2303-5

**Published:** 2017-08-02

**Authors:** Simon Chioma Weli, Ole Bendik Dale, Haakon Hansen, Mona Cecilie Gjessing, Liv Birte Rønneberg, Knut Falk

**Affiliations:** 1 Norwegian Veterinary Institute, P.O.Box 750 Dep., N-0106 Oslo, Norway; 2 Present address: Fiske-liv AS, Marine Harvest Apotekergt. 9A, 6004 Ålesund, Norway

**Keywords:** Gut infection, Gill histopathology, *Salmo salar* L., Chitin staining, Calcofluor-white staining, in situ hybridization

## Abstract

**Background:**

In September 2008, a disease outbreak characterized by acute, severe gill pathology and peritonitis, involving the gastrointestinal tract, was observed in an Atlantic salmon (*Salmo salar* L.) farm in north-western Norway. During subsequent sampling in November 2008 and January 2009, chronic proliferative gill inflammation and peritonitis was observed. Cumulative mortalities of 5.6–12.8% and severe growth retardation were observed. Routine diagnostic analysis revealed no diseases known to salmon at the time, but microsporidian infection of tissues was observed.

**Methods:**

To characterize the disease outbreak, a combination of histopathology, in situ hybridization (ISH), chitin, calcofluor-white (CFW) staining, and real-time PCR were used to describe the disease progression with visualization of the *D. lepeophtherii* stages in situ.

**Results:**

The presence of the microsporidian *Desmozoon lepeophtherii* was confirmed with real-time PCR, DNA sequencing and ISH, and the parasite was detected in association with acute lesions in the gills and peritoneum. ISH using a probe specific to small subunit 16S rRNA gene provided an effective tool for demonstrating the distribution of *D. lepeophtherii* in the tissue. Infection in the peritoneum seemed localized in and around pre-existing vaccine granulomas, and in the gastrointestinal walls. In the heart, kidney and spleen, the infection was most often associated with mononuclear leucocytes and macrophages, including melanomacrophages. *Desmozoon lepeophtherii* exospores were found in the nuclei of the gastrointestinal epithelium for the first time, suggesting a role of the gastrointestinal tract in the spread of spores to the environment.

**Conclusions:**

This study describes the progression of *D. lepeophtherii* disease outbreak in an Atlantic salmon farm without any other known diseases present. Using different methods to examine the disease outbreak, new insight into the pathology of *D. lepeophtherii* was obtained. The parasite was localized in situ in association with severe tissue damage and inflammation in the gills, peritoneal cavity and in the gastrointestinal (GI) tract that links the parasite directly to the observed pathology.

## Background

Atlantic salmon (*Salmo salar* L.) is the most important species for the aquaculture industry in Norway as well as in many other countries [1]. Atlantic salmon production in Norway suffers from several specific diseases that include pancreas disease (PD), heart and skeletal muscle inflammation (HSMI) and cardiomyopathy syndrome (CMS), all of which are characterized by chronic inflammation [2]. Chronic peritonitis is omnipresent due to the general use of oil-adjuvanted vaccines injected intraperitoneally, and this may also elicit autoimmune reactions [3]. Inflammation of the gastro-intestinal (GI) tract is seldom reported from farmed Atlantic salmon, but plant feed components used in brood fish production have been associated with an inflammatory bowel disease - colon cancer-like disease (IBD-CCD) [4]. Diseases affecting the gills are a growing problem in Atlantic salmon farming and several infectious agents have been associated with these diseases [5–7]. However, a lack of culture techniques and experimental models makes it difficult to identify primary pathogens, opportunistic and commensal agents in the plethora of microorganisms present in the gills [6–13].

Microsporidians are obligate, spore-forming intracellular parasites of vertebrates and invertebrates, including fish [14–18] and they are widely distributed in estuaries, sea and fresh water. They are thought to enter their host most often via the gut, injecting the sporoplasm into the host intestinal epithelial cells, spreading systemically by infected, circulating monocytes-macrophages [19, 20]. The spores shed to the environment are very resistant and complex life-cycles involving several host species occur, some even crossing the boundary of cold- and warm-blooded animals including humans [21]. Microsporidians known to cause disease especially in farmed salmonid fish comprise *Loma salmonae*, *Nucleospora salmonis* and *Desmozoon lepeophtherii* [19, 20]. In wild fish, *Nucleospora cyclopteri* has been shown to cause disease in lumpfish (*Cyclopterus lumpus* L.) [22]. *Loma salmonae* has a direct life-cycle, infects orally, and after hematogenic spread, xenomas form in the gills. When xenomas burst, a severe gill inflammation is incited that leads to significant disease losses in farmed*,* Chinook salmon (*Oncorhynchus tshawytshca* Walbaum*)* and rainbow trout (*Oncorhynchus mykiss* Walbaum) [19, 20, 23, 24]. *Nucleospora salmonis* infects a broad range of salmonid fish species and the replication takes place intra-nuclear especially in lymphoblast cells. In clinical cases of Chinook salmon and rainbow trout, anemia and a leukemia-like condition have been observed in which infected lymphoblast infiltrates many tissues and cause enlargement of the kidney, spleen and lower intestine [25]. The disease caused by *N. cycloptera* in lumpfish closely resembles the disease in salmonid fish caused by *N. salmonis* [22]. The *Nucleospora* species are closely related to *D. lepeophtherii,* which was first described by Freeman and others as an infection of salmon sea lice (*Lepeophteirus salmonis* Krøyer) [26, 27] and later in Atlantic salmon [28]. In Atlantic salmon *D. lepeophtherii* has been reported using the junior synonymous name *Paranucleospora theridion* [27]. In sea lice, *D. lepeophtherii* form large xenomas and appear pathogenic to the sea lice [28]. In Atlantic salmon, *D. lepeophtherii* do not form xenomas, but two reproductive cycles have been described by Nylund et al. [29]. In the cytoplasm of leukocytes, macrophages, endothelium and epithelial cells of the skin and gill, many small auto-infective spores (*c.*1.0 μm) are produced and disseminated systemically. Another reproductive cycle takes place in the nucleus of epithelial cells of the skin and gill and results in a few, thick walled spores (*c.*2.0 × 2.5 μm) that are presumably released to the environment [29]. In 2005, Kvellestad and co-workers [13] suggested that the etiology of proliferative gill inflammation (PGI) is multifactorial. In 2010, Steinum and co-workers [6] found that Atlantic salmon with severe PGI appear to have a higher load of *D. lepeophtherii* infection than non-PGI fish. A similar conclusion was reached in an extensive PCR screening of *D. lepeophtherii* infection in farmed Atlantic salmon suffering from a set of different disease conditions [10]. No clear correlation between the diseases and the levels of D. lepeophtherii infection was found, but the *D. lepeophtherii* infection was often present and could possibly aggravate the various disease conditions.

In the present study, we examined fish at three time points during a natural disease outbreak of a severe *D. lepeophtherii* infection, with the aim of addressing the lack of suitable in situ methods and *D. lepeophtherii* disease characterization. To do this, we used a combination of traditional histopathology, in situ labelling and PCR. By comparing the different methods for detection and characterization of *D. lepeophtherii* disease, we uncover new information about *D. lepeophtherii* disease pathogenesis.

## Methods

### Fish sampling

In August 2008, an acute disease problem started affecting fish with normal size and shape weighing about 400 g on the average. The farm is located within a fjord and the nearest representative location for environmental measurements operated by the Norwegian Institute of Marine Research (IMR) is Sognessjøen. In the relevant period of 2008, the measured monthly temperature was higher than normal. The measured temperatures at 1 m depth and the difference from corresponding historic averages from 1935 to 1993 were as follows: May: 11.1 (+2.9) °C; June: 14.2 (+2.5) °C; July: 16.0 (+2.0) °C; August: 16.7 (2.3) °C; September: 15.0 (+2.0) °C; October: 11.6 (+0.9) °C; November: 10.1 (+0.9) °C; December: 7.6 (+0.4) °C; and January 2009 6.1 (-0.1) °C. In a fjord system the surface water are influenced by fresh water and salinity fluctuate around 30‰. The salinity at Sognessjøen in 2008 was predominantly within the normal range around this value.

The 21 diseased fish chosen for further examination in this study were sampled in September 2008 (A1-7), November 2008 (B1-8) and January 2009 (C1-6). Tissue samples were fixed in 10% phosphate buffered formalin and RNAlater (Sigma-Aldrich, Hamburg, Germany) and submitted to the Norwegian Veterinary Institute Oslo for further diagnostic investigations.

### Verification of *D. lepeophtherii* by PCR

A similarly-sized small piece of kidney and/or gill tissue was cut off and lysed overnight at 56 °C in 100 μl of Mole® lysis buffer and 10 μl of Proteinase K solution (Sigma-Aldrich, Hamburg, Germany). DNA from each piece was then extracted on a GenMole (Mole Genetics, Oslo, Norway) using the Mole Genetics DNA Tissue Kit and the DNA tissue protocol. Approximately 900 bp of rDNA was amplified using the primers and protocol of Freeman et al. [22] as described below. All PCR reactions were done using Illustra PuReTaq Ready-To-Go ™ PCR Beads (GE Healthcare, Oslo, Norway). Each reaction contained 1 μl of the forward primer, 1 μl of the reverse primer, 3 μl of the template DNA and 20 μl of sterile water. PCR products were sent to Macrogen for sequencing (Macrogen, Amsterdam, The Netherlands). All products were sequenced using the PCR primers. Sequences were proofread in VectorNTI ver. 11 (Invitrogen, Oslo, Norway) and subjected to a GenBank BLASTn search to search for identity with known sequences. The same samples were also subjected to RT-PCR analyses following the protocol of Nylund et al. [29], but targeting the rRNA gene and each analysis was repeated once. Average values for replicates were calculated.

### Histopathology

The formalin-fixed tissues were paraffin-embedded and serial sections stained with hematoxylin and eosin (HE) according to standard histological techniques [30] and the in situ methods described below.

### Calcofluor White (CFW) method

CFW staining was performed according to Monheit and others and Stine et al. [31, 32]. Briefly, paraffin-embedded tissue sections were de-waxed in xylene, rehydrated through graded alcohols and washed in distilled water. Subsequently, slides were treated with a 10% KOH solution in distilled water for 2 min and stained with 0.05% Calcofluor white stain (Becton Dickinson, Franklin Lakes, USA) for 2 min. The stained samples were immediately examined with epi-fluorescence microscopy for the presence of spores.

### Fluorescent chitin-binding probe (FCP) method

The chitin-binding lectin probe made from chitinase A1 of *Bacillus circulans* was used for detection of *D. lepeophtherii* [33]. After de-waxing tissue sections in xylene and rehydration through graded alcohols, sections were washed in distilled water and subjected to antigen retrieval by microwave treatment in citrate buffer solution (0.01 M, pH 6.0). Non-specific binding sites were blocked by incubation in 5% fetal bovine serum (FBS) for 20 min at room temperature. Fluorescein-conjugated chitin-binding lectin probe (New England BioLabs, Hitchin, UK) diluted 1:100 in PBS in 1% FBS was then added onto each tissue section and incubated overnight in dark at room temperature. Sections were washed in PBS, mounted with SlowFade Gold anti-fade reagent (Invitrogen, Oslo, Norway) and examined by epi-fluorescence microscopy (Leica, DM5000B).

### In situ hybridization (ISH) method

#### RNA isolation

RNA from *D. lepeophtherii*-infected samples was isolated with Trizol™ reagent (Life Technologies Burlington, ON, Canada) and quantified using the NanoDrop ND-100 spectrophotometer (NanoDrop Technologies, Wilmington, USA). *Desmozoon lepeophtherii* was amplified using a SuperScript III Platinum One-Step Quantitative RT-PCR system. Total reaction of 50 μl containing 1 μg of total RNA, 25 μl 2× reaction mix, 1 μl Platinum *Taq* enzyme and 10 pmol of primer sets (forward: 5′-GTC TGT GGA TCA AGG ACG AA-3′; reverse: 5′-ACT GAT ATG CTT AAG TTC AGG-3′; GenBank accession no. AJ431366) previously described by Freeman et al. [22], was used. The thermocycling conditions were 30 min at 60 °C reverse transcription, 3 min of denaturation step at 95 °C and 30 amplification cycles (95 °C for 30 s, 55 °C for 30 s and 72 °C for 1 min) and a 10 min elongation step at 72 °C. PCR product of approximately 800 bp was amplified and visualized in ethidium bromide-stained 1.2% agarose gel.

#### Probe preparation

The amplified PCR product was purified using the Nucleospin extract II (Macherey-Nagel GmbH, Düren, Germany), cloned into *Escherichia coli* cells (pGEM® - T Vector Systems; Promega, Fitchburg, WI, USA) and plasmid DNA was purified using Nucleospin® Plasmid (Macherey-Nagel GmbH, Düren, Germany). For verification of template, plasmid DNA with insert was sequenced with primers for *D. lepeophtherii* and M13 and *D. lepeophtherii* sequence downloaded from GenBank (accession no. AJ431366) was included in the alignment to verify the identity of *D. lepeophtherii*.

#### Digoxigenin-labelled RNA probes

Purified plasmid DNA was digested overnight at 37 °C with restriction enzymes *Hind*III and *Pvu*II (Promega, Fitchburg, WI, USA). Probe-labelling was performed using DIG RNA Labelling kit (SP6/T7) as recommended by the manufacturer (Roche Diagnostics, Oslo, Norway) to generate DIG-labelled RNA anti-sense and sense probes, respectively. Labelled RNA was purified using illustra™ probe Quant™ G-50 μ columns probe purification kit (GE Healthcare, Oslo, Norway) according to the manufacturer’s instructions.

#### In situ hybridization

Incubations were performed at room temperature unless otherwise stated. Serial sections were incubated with anti-sense and sense probes as positive and negative controls, respectively. Hybridization was performed according to Solstad et al. [34]. Briefly, tissue sections were equilibrated in DEPC-PBS, deparaffinised, rehydrated and permeabilized in 0.3% Nonidet P-40 (Roche Diagnostics, Oslo, Norway) in DEPC-PBS. Sections were digested in 10 μg/ml proteinase K in TE buffer (50 mM HCL, 5 mM EDTA, pH 7.5) for 10 min at 37 °C, post-fixed for 10 min in 4% formaldehyde in DEPC-PBS and washed in DEPC-PBS. Sections were acetylated in 150 ml DEPC-water containing 750 μl acetic acid anhydride and 2 ml triethanolamine for 10 min. Pre-hybridization was performed at 55 °C for 1 h in a humidified chamber with hybridization buffer containing 50% formamide, 2× SSC, 50%, dextran sulphate (*w*/*v*), 4 mg/ml Calf Thymus DNA, and 20 μl 10× Denhardts solution. Hybridization was performed using 40 ng anti-sense and sense probes as positive and negative control at 55 °C in a humidified chamber overnight. Washing was performed at 55 °C with 2× SSC, 1× SSC, 0.5× SSC, 0.1× SSC and TBS buffer (0.1 M Tris-HCl, pH 7.5; 0.15 M NaCl for 5 min). Immunodetection of the *D. lepeophtherii* probe was performed with alkaline phosphatase-conjugated anti-DIG Fab fragments (Roche Diagnostics) using Fast Red TR/Naphthol As-MX substrate (Sigma-Aldrich, Hamburg, Germany).

## Results

### Case description including clinical, gross pathological findings

**Fig. 1:**
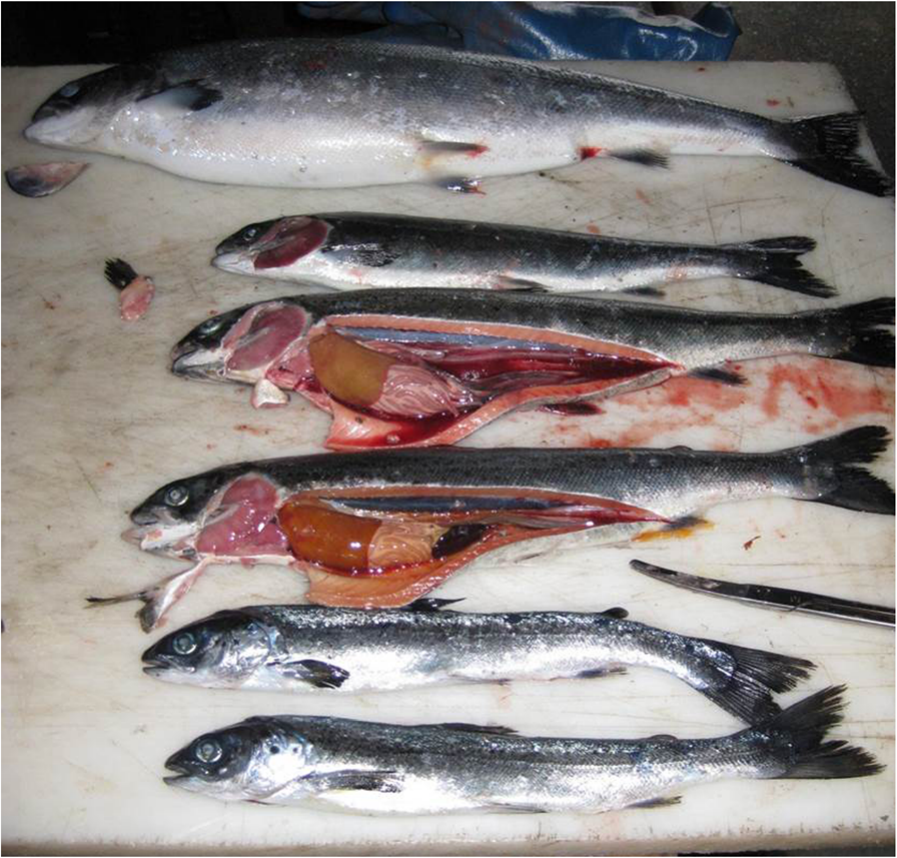
Autopsy showing the degree of stunted growth: the fish on the top has grown normally

The sea-water farm at the Norwegian west coast stocked the affected year class of Atlantic salmon smolts during spring 2008. The fish had been given intraperiotoneal injection with an oil-adjuvant vaccine. After sea-water transfer, the only health problem was the presence of some emaciated fish that was attributed to a previous outbreak of infectious pancreatic necrosis (IPN) during the freshwater stage. In August 2008, an acute disease problem started affecting fish in normal size and shape weighing about 400 g on average. The daily mortality was highly variable and from seawater transfer to the end of January 2009 the cumulative mortality ranged from 5.6 to 12.8% in the 8 net-pens involved. The clinically affected fish showed stunted growth and weighed 20–25% of normal growing fish by the end of January 2009. The clinically affected fish swam sluggishly, but responded with flight upon netting. They had stopped eating and the skin often appeared dark with a greenish hue. On autopsy the gills appeared uneven and with more mucus than normal. Some fish had small skin bleedings at the fin bases (Fig. 1). In the abdomen variable liver color, swollen spleen, ascites, hyperemia and often peritonitis as with high grade vaccination side effects were noted. Also a few fish had greyish, cheese-like enlarged kidney (Fig. 1).

### Real time PCR

Kidney and/or gill samples from 20 fish from the 3 different samplings were analyzed and 20 out of 21 fish were positive for *D. lepeophtherii* (Table 1)*.* From one fish (A2) there was no PCR result, but *D. lepeophtherii* was found with ISH. Sequences (~800 bp) were obtained from most individual samples by conventional PCR. Apart from two ambiguous bases, the Blast search showed that the sequences were identical to *D. lepeophtherii* (AJ431366) confirming the identity of the parasite. Thus all fish were infected with *D. lepeophtherii*. Non-normalized PCR threshold cycles among tested samples ranged from 16 to 31. The lowest Ct-values were found in the first sampling. When relating the Ct-values to the pathology stages observed, the acute stage samples were represented by Ct-values from 20 and below. The Ct values found in the non-acute stages were from 20 and above, with considerable variation and overlap between the sub-acute and chronic stages (Table 1).

**Table 1:** Comparing of semi-quantitative scoring of sampled fish tissues based on HE staining, calcofluor white staining and real-time PCR Ct-values

Pathology scores of each fish	Fish ID	Histopathology scores			Calcofluor staining scores		Real-Time PCR Ct-values	
		Gill Microvesicles	PGI	Peritonitis	Gill	Peritoneum	Heart/Kidney	Gill
Acute	A6	3.0	1.0	2.0	3.0	2.0	17	nd
Acute	A1	3.0	1.0	1.0	2.0	2.5	17	nd
Acute	A3	3.0	1.0	2.0	3.0	1.5	17	nd
Acute	A7	3.0	1.0	3.0	2.0	3.0	18	nd
Acute	A4	3.0	1.0	1.5	3.0	1.0	20	nd
Acute	B1	3.0	1.0	2.5	3.0	1.0	nd	20
Subacute	B2	3.0	2.0	1.5	3.0	1.0	nd	22
Subacute	A5	2.0	1.0	3.0	1.0	1.5	24	nd
Subacute	B3	2.0	2.0	3.0	1.5	3.0	nd	27
Subacute	A2	1.0	1.0	3.0	1.0	0.5	nd	nd
Chronic	C2	2.0	2.5	1.5	2.0	1.0	21	20
Chronic	B4	2.0	3.0	2.5	2.5	2.0	nd	23
Chronic	C4	1.0	2.0	3.0	1.0	1.5	26	24
Chronic	C3	1.5	2.0	2.0	1.0	1.0	28	25
Chronic	B8	2.0	1.0	2.0	1.0	1.5	nd	26
Chronic	C1	1.0	0.5	1.5	1.0	0.0	25	26
Chronic	C6	1.0	2.0	1.5	0.0	0.0	23	27
Chronic	B7	1.0	0.5	3.0	1.0	2.5	nd	28
Chronic	B6	1.0	0.5	2.0	1.0	0.0	nd	30
Chronic	B5	1.0	0.5	2.0	1.0	1.0	nd	30
Chronic	C5	1.0	1.0	2.0	1.0	1.5	26	31

*Abbreviation: nd* not done

### In situ detection methods with CFW, FCP and ISH

**Fig. 2:**
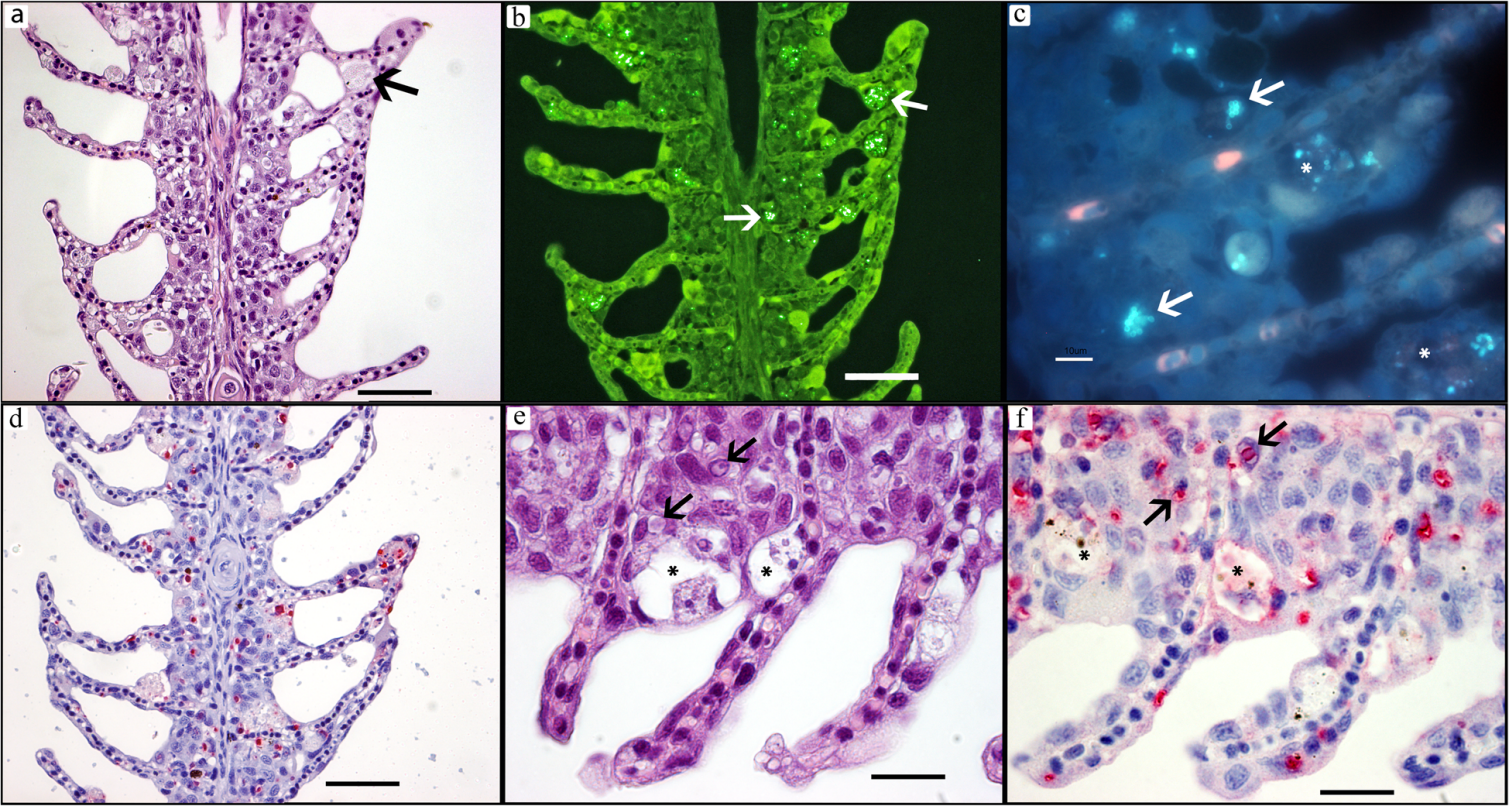
Acute group: gill infection co-localized with necrosis. **a** HE-stain, distal adherences between slender lamellas with swollen, necrotic cells sometimes with poorly stained, iridescent *D. lepeophtherii* spore clusters (*arrow*). **b** FCP, green FITC chitin staining of *D. lepeophtherii* spore clusters (*arrows*) corresponding with the iridescent spores on HE-stain in **a**. **c** CFW, UV-blue chitin fluorescence of *D. lepeophtherii* spore clusters (*arrows*), necrosis (*). **d** ISH, rRNA probe staining proliferative stages of *D. lepeophtherii *red. **e** HE-stain, severely infected gill epithelium, swollen necrotic cells (*), cytoplasmic and intranuclear proliferative stages surrounded by a clear halo (*arrows*). **f** ISH, same gill as (**e**), intense staining of the halo surrounded bodies in both cytoplasm and nucleus (*arrows*), less staining in necrotic cells often with some melanin granules (*). *Scale-bars*: **a**, **b**, **d**, 50 μm; **c**, **e**, **f**, 10 μm

*Desmozoon lepeophtherii* spores on HE were difficult to observe and appeared as unstained, iridescent granules (Fig. 2a). These observations were in contrast to FCP, which revealed many fluorescent spores in bright green (Fig. 2b) and bright blue with CFW (Fig. 2c). The FCP gave stronger signal that was easier to differentiate from the tissue background than CFW. In the cytoplasm, small (*c.*1 μm) round, thin-walled spores in clusters were found that are regarded auto-infective. Fewer, larger (*c.*3–4 μm) oblong spores was occasionally found, these are formed in the nucleus and released to the environment [29]. ISH did not react notably with spores, but reacted strongly with cytoplasmic and intra-nuclear round bodies of variable size often surrounded by a clear halo (Fig. 2d and f). No staining was observed with sense probes used as negative controls. The ISH stained bodies could be recognized as uniformly pink on HE-stained preparations, but stood out from the surrounding cytoplasm or nucleus only due to the halo. The stages stained by the rRNA probe may represent ribosome-rich stages like merogonia and plasmodia [29].

### Histopathology

**Fig. 3:**
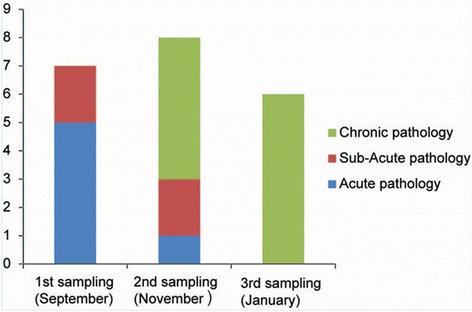
Number of fish with acute, sub-acute or chronic histopathology at the three different sampling time points. The acute stage had much necrotic gill lesions with numerous *D. lepeophtherii* that were also systemically disseminated. The sub-acute is an intermediate stage defined by many foamy macrophages and little necrosis with *D. lepeophtherii*, but also with features in common with chronic stage as gill epithelial proliferation and granulomatous inflammation. The chronic group were separated from the sub-acute group by the presence of reparative changes like fibrin and collagen deposits in the granulomatous peritonitis and more gill epithelial proliferation

As expected in a natural disease outbreak the disease course and histopathological lesions were not synchronous in fish sampled at the same time point. However, there was a general progression from acute to chronic changes over time. We grouped and described the sampled fish according to their predominating histopathology as acute, sub-acute or chronic histopathology (Fig. 3).

### Overview of histopathological findings

The acute group had necrosis especially in the gills and peritoneal cavity with severe, disseminated *D. lepeophtherii* infection that was as also found in most other organs studied. The number of *D. lepeophtherii* seen in the lesions was low in both sub-acute and chronic groups compared to the acute group, but a few necrotic foci with more *D. lepeophtherii* were occasionally found. The complex pathology in both acute and chronic features, suggest a persistent infection or reinfections that triggers the chronic inflammation. Proliferation of inter-lamellar gill epithelium increased from very sparse in the acute to a filling of the inter-lamellar space that could be complete in the chronic group. No pathology suggesting other known salmonid diseases, including IPN, were found.

### Histopathological details relating to acute, sub-acute and chronic stage disease

#### Acute stage

**Fig. 4:**
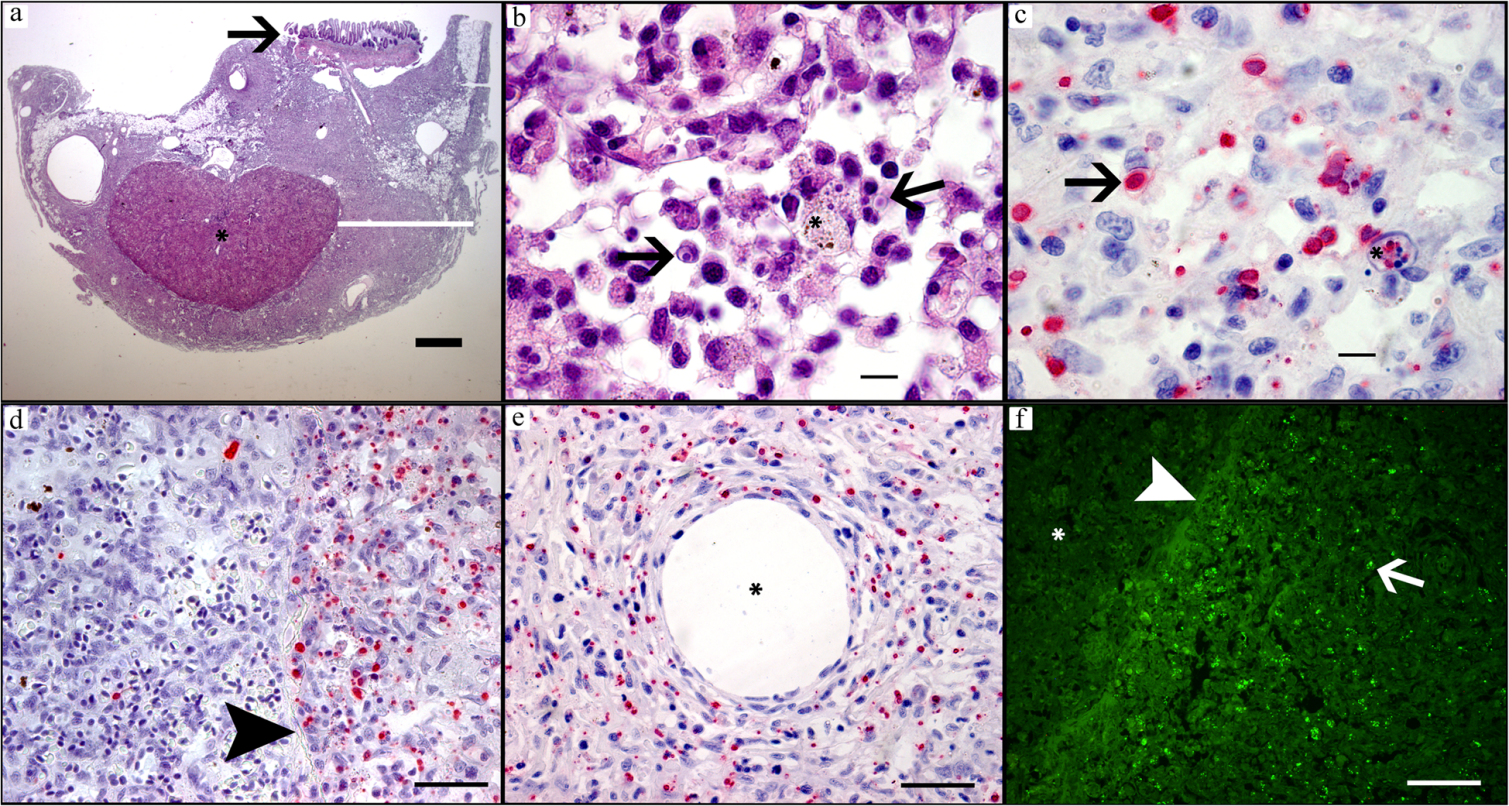
Abdominal cavity with spleen and severe *D. lepeophtherii* infection in both a diffuse acute peritonitis and chronic vaccine related lesions. **a** HE-stain, spleen (*) and intestine (*arrow*) and perivisceral inflammatory tissue (*white bar*). **b**-**f** show details of **a**: **b** HE-stain of perivisceral inflammatory tissue with necrosis and iridescent spores (*) and pink proliferative stages of *D. lepeophtherii* surrounded by a halo (*arrows*). **c** ISH of same area as **b** showing proliferative stages of *D. lepeophtherii* stained red (*arrow*) including in a necrotic cell (*). **d** ISH staining proliferative stages of *D. lepeophtherii* red in the border area of spleen and perivisceral inflammatory tissue, many in the peritonitis, a few in the spleen, arrowhead pointing at the spleen capsule. **e** ISH staining proliferative stages of *D. lepeophtherii* red both in the diffuse peritonitis and in the organized, circular encapsulation of a droplet (*), presumably vaccine in oil-adjuvant. **f** FCP of border area of spleen and perivisceral inflammatory tissue showing green fluorescent *D. lepeophtherii* spores (*arrow*) especially in the peritonitis, while the spleen (*) appear less infected borderline between spleen and peritoneum (*arrowhead*). *Scale-bars*: **a**, 1 mm; **b**, **c**, 10 μm; **d**-**f**, 50 μm

**Fig. 5:**
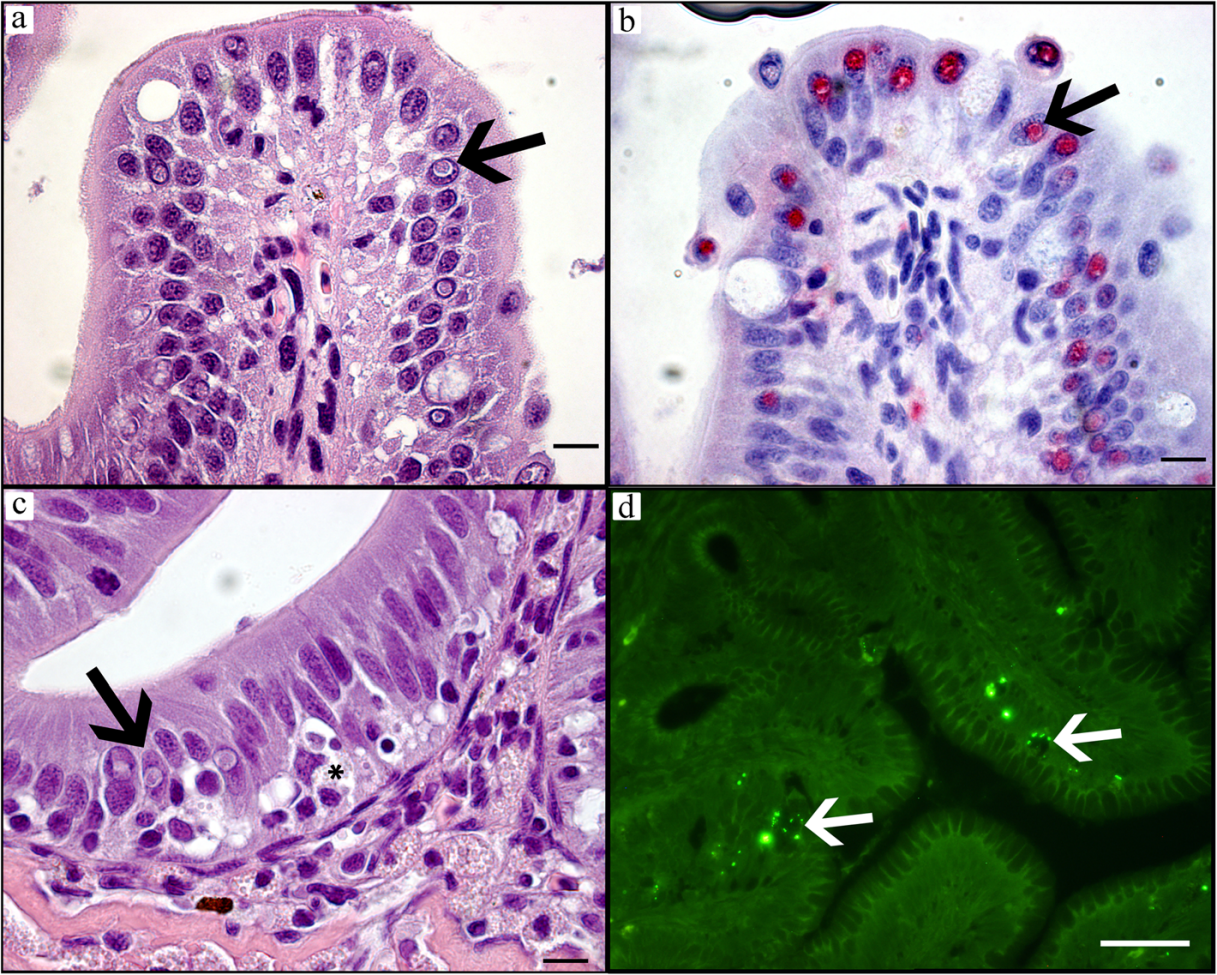
*Desmozoon lepeophtherii* infection of the gastro-intestinal tract. **a** HE-stain, many intranuclear proliferative stages surrounded by a halo (*arrow*) in hind gut epithelium. **b** ISH of same hind gut area as in (**a**) with the intranuclear proliferative stages stained red (*arrow*). **c** HE-stain of pyloric ceca epithelium with necrosis (*) and intranuclear proliferative stages surrounded by a halo (*arrow*). **d** FCP showing green fluorescent *D. lepeophtherii* spore clusters (*arrows*) in the stomach epithelium. *Scale-bars*: **a**-**c**, 10 μm; **d**, 50 μm

In acute stages the gill epithelium was severely infected and appeared “dotted” due to widespread necrosis, swollen cells and morphologically different stages of *D. lepeophtherii.* Clusters of very small *D. lepeophtherii* spores in swollen cells were poorly stained, slightly iridescent in HE sections (Fig. 2a), but strongly fluorescent by both FCP (Fig. 2b) and CFW (Fig. 2c). Proliferative stages of *D. lepeophtherii* formed many cytoplasmic and some intra-nuclear, roundish, variably-sized pink bodies discernible on HE due to a clear halo (Fig. 2a and e), while strongly stained with ISH (Fig. 2d and f). Focal necrosis and micro-vesicles containing debris and various microsporidian stages were variably stained by the in situ methods (Fig. 2d, f and *). The host response appeared limited to some infiltrating, often swollen, infected macrophages. Infected epithelial cells were involved in some lamellar adhesions (Fig. 2a), but the inter-lamellar spaces were largely intact as the gill epithelium had not proliferated, but moderately thickened due to swollen, infected cells. No other pathogens were found except for a few epitheliocysts superficially in the lamellar epithelium of three fish. As expected, all six fish had few well-organised granulomas around vacuoles with intraperitoneally injected oil adjuvant vaccine which is common for farmed Atlantic salmon. In addition four fish had a diffuse, acute peritonitis with necrosis and severe *D. lepeophtherii* infection as described for gill epithelium except that the intra-nuclear *D. lepeophtherii* stage was not observed (Fig. 4). In the two most severe cases, the peritonitis was partly continuous with similar lesions within the adherent GI-walls of stomach, pyloric ceca and hind gut segments. The severity of these lesions diminished towards GI-lumen, often appearing to stop at the stratum compactum. In the lamina propria there was in general a diffusely distributed, sparse cytoplasmic infection and focal necrosis with no apparent link to the peritonitis. In the two most severely infected fish, widespread intra-nuclear infection of the gut epithelium out-numbered by far this *D. lepeophtherii* stage in the gill epithelium (Fig. 5a and b). Necrotic and degenerate cells often enlarged with spores or proliferative stages were found especially in the spleen stroma, kidney interstitial and endothelial cells of the heart bulbus arteriosus (data not shown). The infected cells appeared to be macrophages, endothelial and lymphopoietic or hematopoietic cells. Some infected cells found intravascularly suggested a hematogenic spread of *D. lepeophtherii*. The livers had some degenerative changes that could not be related directly to the *D. lepeophtherii* infection, but some focal necrosis and infections with *D. lepeophtherii* were observed in and around vessel walls. In the skin, a few necrotic dermal cells with halo surrounding the proliferative stages stained by ISH were observed in one fish only. The number of melanomacrophages appeared slightly or moderately increased in many tissues.

#### Sub-acute stage

**Fig. 6:**
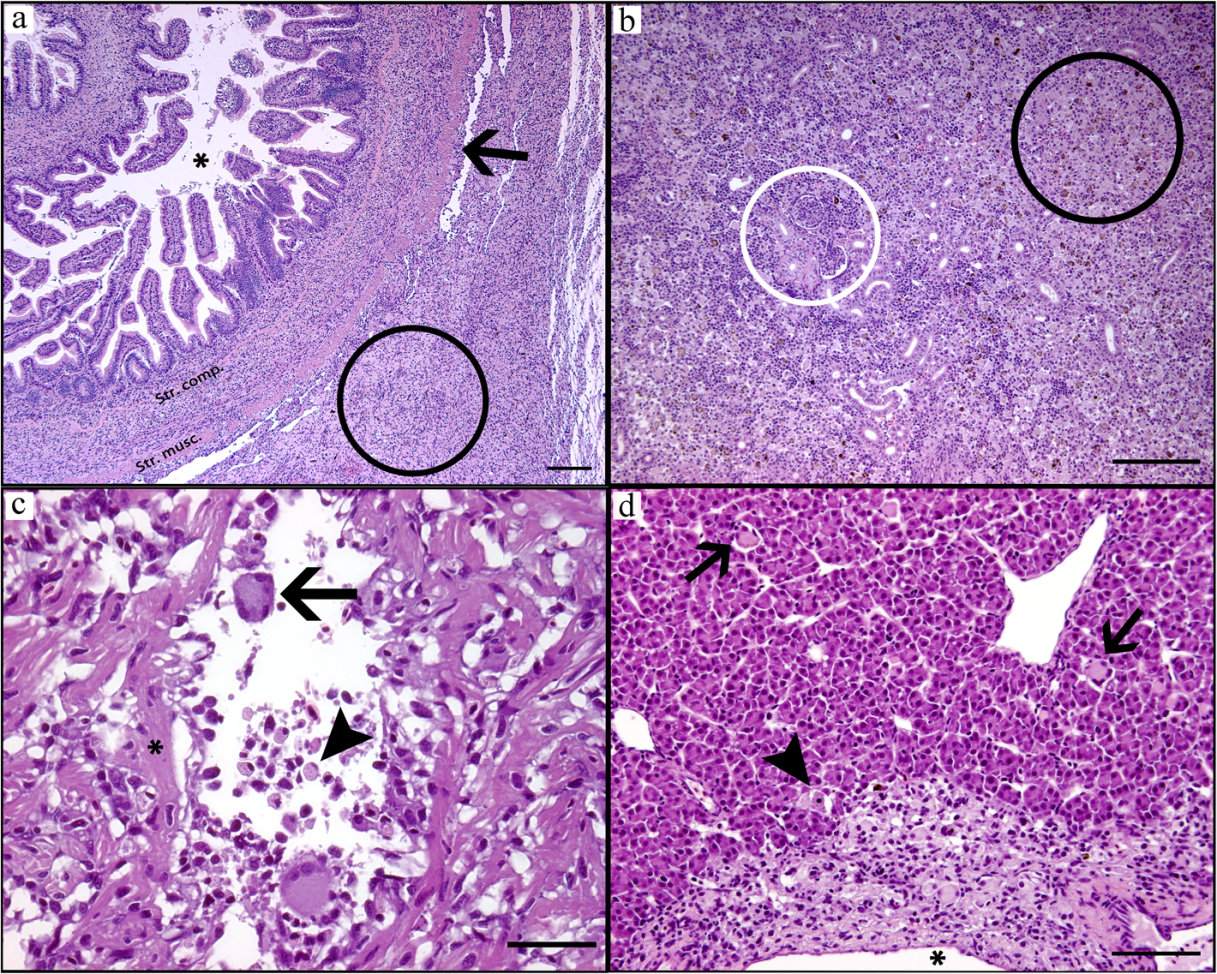
Sub-acute pathology with foamy macrophage infiltration in hind gut, peritoneum, kidney, heart and liver. **a** HE-stain of hind gut with lumen (*), serosa (*arrow*) and peritonitis (*circle*) dominated by large foamy macrophages that also infiltrate the gut wall as deep as the stratum compactum, **b** HE-stain of kidney with foamy macrophages (*black circle*) largely replacing the normal tissue (*white circle*). **c** HE-stain of heart lumen with foamy macrophages, including large multinucleated giant cells (*arrow*), eosinophilic granular cells (*arrowhead*) and endocarditis with some degenerative muscle changes (*). **d** HE-stain of liver, vasculitis with many foamy macrophages, lumen of vessel (*) at bottom of picture, in the liver parenchyma foamy giant cells (*arrows*) and some necrosis (*arrowhead*). *Scale-bars*: **a**, 200 μm; **b**, **d**, 100 μm; **c**, 50 μm

The sub-acute stage was dominated with substantial host cellular response and lower levels of visible *D. lepeophtherii*. On autopsy two fish (A2 and A5) had “cheese-like” tissue in the abdomen and kidney that consisted of masses of foamy macrophages, sometimes multinucleated. The peritonitis appeared “invasive” extending deep into adjacent GI-walls destroying the muscular layers, stratum compactum and sometimes also the lamina propria while the epithelium was mostly unaffected (Fig. 6a). Two of these fish had large infiltrations of foamy macrophages in organs including spleen, kidney (Fig. 6b), heart (Fig. 6c), liver (Fig. 6d), gill and dermis of the skin. The hearts had extensive epi- and endocarditis with many foamy macrophages and eosinophilic granular cells. The liver had severe vasculitis with foamy macrophages. Two other fish with sub-acute disease had less infiltration with foamy macrophages and more visible stages of *D. lepeophtherii*. Erythrophagocytosis and variable hemosiderosis were seen in the spleens. In the gills there was some necrosis as in the acute stage, but also proliferation of epithelium that often filled about half of the inter-lamellar space. Other pathogens found in the gills were limited to a few epitheliocysts in one fish, and some *Trichodina* spp. ciliates in another fish.

#### Chronic stage

**Fig. 7:**
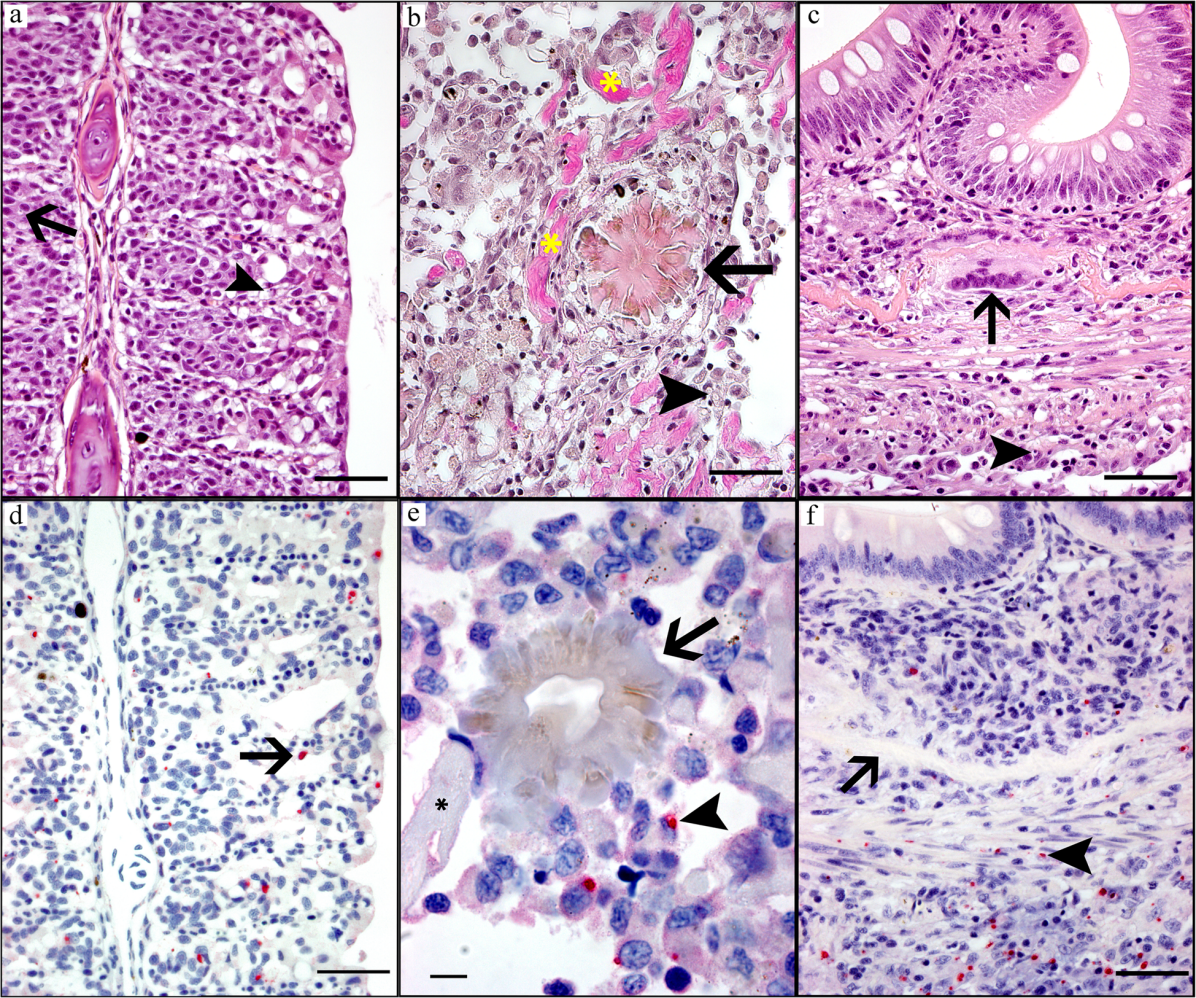
HE, van Gieson and ISH stained sections showing chronic pathology in gill, peritoneum and intestine. **a** HE-stain of gill with extensive epithelial proliferation filling the space between lamellas and some cytoplasmic *D. lepeophtherii* proliferative stages surrounded by halo (*arrow*) and necrosis (*arrowhead*). **b** van Gieson-stain of granulomatous inflammation and fibrosis in the abdomen staining collagen deposits red (yellow *), *D. lepeophtherii* cytoplasmic proliferative stage (*arrowhead*) and Splendore-Hoeppli concrement (*arrow*). **c** HE-stain of invasive peritonitis in GI-wall, including *D. lepeophtherii* cytoplasmic proliferative stage (*arrowhead*) and multinucleated giant cell (*arrow*). **d** ISH of same area as in **a** showing proliferative stages of *D. lepeophtherii* stained red (*arrow*) in proliferative gill lesions, **e** ISH of same peritoneal area as in **b** showing proliferative stages of *D. lepeophtherii* stained red (*arrowhead*) and Splendore-Hoeppli concrement (*arrow*). **f** ISH of same intestine as in **c** showing proliferative stages of *D. lepeophtherii* stained red (arrowhead) and stratum compactum (*arrow*). *Scale-bars*: **a-d**, **f**, 50 μm; **e**, 10 μm

This stage revealed extensive proliferation of the gill epithelium in 5 fish (Fig. 7a) and granulomatous inflammation and fibrosis (Fig. 7b) in the abdomen of all fish. As in the acute and subacute groups the peritonitis appeared invasive into the GI-walls, but now more granulomatous and giant cells could be found destroying GI-wall elements (Fig. 7c). In proliferative gill (Fig. 7d), abdominal (Fig. 7e) and intestinal (Fig. 7f) lesions, moderate numbers *D. lepeophtherii* were visible. However, in general there were very sparse numbers *D. lepeophtherii* in the chronic lesions, occasional small foci of *D. lepeophtherii* infection with necrosis were encountered. In the fish with no or sparse *D. lepeophtherii* infection there was also little gill pathology. An intra-nuclear infection of the gut epithelium was only found in one fish, and apparently not associated with the “invasive” peritonitis. Other organs had only sparse to moderate inflammation dominated by macrophages, sometimes foamy, and often also some melanomacrophages. The hearts had consistently a sparse to moderate epi- and endocarditis. In the kidney and especially the spleen, some erythrophagocytosis and hemosiderosis were also found. In the liver only a sparse, diffuse distribution of melanomacrophages was common, except in one fish with multiple, zonal parenchymal liver necrosis (C2). This fish also had another gill infection; *Tenacibaculum*-like bacteria were found in distal necrosis of some adjacent filaments. Notably, polymorphic leukocytes and circulatory disturbances including haemorrhages were associated with the *Tenacibaculum*-like bacteria. The other parts of these gills had extensive proliferation of the gill epithelium with a moderate to severe *D. lepeophtherii* infection. Other visible gill pathogens were limited to a few *Trichodina* spp. and superficial epitheliocysts as in the acute and sub-acute groups, but the prevalence appeared higher as this was a finding in all fish of the chronic group.

## Discussion

Previous studies have used PCR and histopathological examination to show the prevalence of *D. lepeophtherii* and its association with gill disease in Norwegian and Scottish Atlantic salmon farms [29, 35]. The paucity of histopathology information about *D. lepeophtherii* infections is probably due to the difficulty of detecting poorly staining, minute spores and nondescript, pleomorphic proliferative stages in tissue sections. In the present study, we have addressed the lack of suitable in situ methods to detect *D. lepeophtherii* by using a combination of histopathology, CFW, FCP and ISH, to describe the disease progression. PCR method was used to confirm *D. lepeophtherii* infections. We used in situ methods for visualization of the *D. lepeophtherii* stages in association with severe tissue damage, and established a direct relationship between the parasite and observed pathology. Since experimental models are currently lacking, we examined a natural disease outbreak in farmed Atlantic salmon and chose a case where the *D. lepeophtherii* infection appeared to be dominant.

We have shown for the first time, replication of *D. lepeophtherii* in the gut epithelium which could be a major source of dissemination of spores to the environment. This suggests that the infection dynamics of *D. lepeophtherii* may be more complex than previously assumed [27, 29]. Generalized and severe infection was observed in clinically diseased fish. The gills and the abdominal cavity seemed to be predilection sites for *D. lepeophtherii* replication that resulted in necrosis and damage to cells including macrophages. Thus, in the acute pathology stages the link between infection and pathology was obvious. In the sub-acute and chronic stages, infection levels in tissue sections were much lower with sparse focal and necrotic lesions. The tissues were also dominated by host responses, including macrophage infiltration, chronic peritonitis and gill hyperplasia. While the initial pathology seems to be caused by *D. lepeophtherii* proliferation, the late pathology may have been due to the severe host responses. This was reflected by change in the gills from the acute necrotic lesions to chronic proliferation of gill epithelium. In the abdomen, micro-vesicles were not formed as surrounding, intact multilayered epithelial tissue are needed to form the vesicle wall. However, necrosis and the halo proliferative stages of *D. lepeophtherii* were evident in the acute stages, and *D. lepeophtherii* infection was confirmed by in situ staining. In contrast, the chronic stage *D. lepeophtherii* pathology in the abdomen was not obvious.

Farmed Norwegian Atlantic salmon are vaccinated intraperitoneally with an oil-adjuvant vaccine before sea-water transfer. *Desmoozon lepeophtherii* infect macrophages [29], which could migrate to inflammatory foci like vaccine-induced granulomas and then infect the same lesions. Thus, any granulomatous inflammation due to *D. lepeophtherii* would be additional granulomas to the pre-existing granulomatous vaccine side-reaction. This may result in co-localization of *D. lepeophtherii* infection and vaccine side-reactions. A characteristic that could distinguish the *D. lepeophtherii* related inflammation was the invasiveness deep into the gastrointestinal muscular walls not expected by an inert, killed vaccine. Moreover, this increasing granulomatous inflammation came at a time when vaccine side-effects are expected to decrease. Thus, in cases of stunted growth and unexpected vaccine side-reactions it would be pertinent to investigate if *D. lepeophtherii* could be involved.

In this study, several methods were chosen to diagnose *D. lepeophtherii* since no single method stains both spores and proliferative stages. The spore staining was chosen as it targeted an inherent trait of the spores: the chitinous endospore wall. The quickest and most generally applicable method was the calcofluor method. The broad specificity is ideal for an initial investigation to uncover all agents with a chitinous wall, including fungi. Although morphology may indicate pathogen identity, sequencing rRNA gene would be necessary to identify these at the species level. The spores can also be stained by Gram [35] or Luna [36], but this will not give any information on the presence of a chitinous spore wall. The fluorescein chitin probe (FCP) is chemically more specific for chitin and yields permanent slides easier to read than CFW. Both CFW and FCP worked well to demonstrate spores in this study, but presently the commercial reagent for FCP utilizing the chitin binding protein of *Bacillus circulans* as probe seems unavailable. These findings correlate with previous reports of high binding affinity and detection of chitin cell walls [37, 38]. A different method was necessary to stain the proliferative stages before spore formation. Since these stages are metabolically active with many ribosomes, we developed an ISH targeting rRNA sequences. The specificity of the probe was confirmed by the use of the sense probe. This procedure allowed a specific diagnosis of *D. lepeophtherii* infection and evaluation of the pathological lesions on the same sections from paraffin embedded tissue by ordinary light microscopy. This method clearly showed that most of the pathology was linked to the proliferative stages of *D. lepeophtherii* and that the halo surrounded bodies on HE sections were indeed *D. lepeophtherii*. Although our procedure was excellent to explore the relationship between the *D. lepeophtherii* and the pathology, simpler procedures, e.g. FISH and using DNA probes could be more practical for routine diagnostic purposes.

Real-time PCR was undoubtedly the most sensitive and specific way to detect the *D. lepeophtherii* infections. The results showed that the in situ methods are less sensitive and that PCR is the method of choice to just determine the presence of the agent. Real-time PCR can also be quantitative or semi-quantitative, but if rRNA was targeted the Ct-values would reflect the number of ribosomes and not individual *D. lepeophtherii* organisms. Spores that are metabolically inactive would contribute little to the Ct-values. We targeted the ribosomal gene, so the Ct-values can reflect the gene, including those in spores. Unfortunately, as our samples for PCR were taken in various ways for general diagnostics, it was not possible to follow the *D. lepeophtherii* infection by PCR systematically over time in specific organs. Nevertheless, the results indicate that acute stages with generalized infection and necrotic lesions with many *D. lepeophtherii* spores present could be associated with Ct-values in the lower range. However, looking at the in situ methods it appears that infection could be very unevenly distributed between organs and variable over the course of the disease. This suggests that PCR data from the disease stages with localized rather than generalized infection should be interpreted with caution. Thus, it remains to be known how a best sample can be obtained for PCR especially in the chronic stages of *D. lepeophtherii*-related disease. Further, PCR data would always benefit from being interpreted in the light of pathology investigations, and here these suggest that the sensitized host may respond with severe chronic inflammation to even low infection levels.

We used the term acute pathology in the description of the necrotic lesions with little host response and severe *D. lepeophtherii* infection. However, this high level of infection may have developed over time prior to the disease level found on our first sampling. We do not know why this level of infection had built up, but the clinical disease history and our findings may provide some clues. Although not causing problems, sea-lice were present and source of the *D. lepeophtherii* infection. On the first sampling we found the highest level of *D. lepeophtherii* intranuclear infection in the intestine of emaciated fish due to the freshwater IPN outbreak. This intra-nuclear stage of *D. lepeophtherii* gives rise to the resistant spores released to the environment. Gill and skin epithelium have earlier been reported as the cell type shedding spores [10, 29]. However, the intra-nuclear intestinal infection was more severe than in gill or skin tissue, suggesting that the gut can be very important for the dissemination of *D. lepeophtherii*. The large number of fish in close confinement in a sea-water salmon farm environment may facilitate a fecal-oral transmission route other microsporidia are known to exploit [39]. In other intra-nuclear fish microsporidia, *Nucleospora salmonis* [25, 40] and *Enterospora nucleophila* [41], the gut is involved. Clearly the complex life-cycles and diseases linked to *D. lepeophtherii* need to be explored further. The economic loss was significant in this disease outbreak due to a moderate mortality, and more importantly, the severely stunted growth of many fish. It is noteworthy that most gill diseases are multifactorial and resolving the roles of a growing number of infectious agents will require extensive experimental and epidemiological investigations. We hope that the methods and observations from this case study of a *D. lepeophtherii* infection will contribute to this process.

## Conclusions

The economic impact of gill disease to Atlantic salmon farms annually is huge. *Desmozoon lepeophtherii* has previously been found to be associated with gill disease in Atlantic salmon. However, information about the pathology associated to different stages of the parasite over the course of infection is limited. Here we used different in situ diagnostic method to associate the proliferative stages of *D. lepeophtherii* with necrosis in the acutely diseased fish. In chronically diseased fish with stunted growth we report lower levels of infection associated with severe inflammatory reactions, which revealed the importance of host responses to the pathology. We also described intranuclear infection of the gut epithelium, which implicated the gut for the first time as a source of *D. lepeophtherii* spore shedding to the environment. Our results provide evidence in favour of combining different diagnostic tools to better understand disease pathology in fish.

## Abbreviations

CFW: Calcofluor-white
CMS: Cardiomyopathy syndrome
DEPC: Diethylpyrocarbonate
FCP: Fluorescent chitin probe
GI: Gastrointestinal
HE: Hematoxylin and eosin
HSMI: Heart and skeletal muscle inflammation
IBD-CC: Inflammatory bowel disease - colon cancer-like disease
IPN: Infectious pancreatic necrosis
ISH: In situ hybridization
PD: Pancreas disease
PGI: Proliferative gill inflammation
